# Machine learning estimation of tissue optical properties

**DOI:** 10.1038/s41598-021-85994-w

**Published:** 2021-03-22

**Authors:** Brett H. Hokr, Joel N. Bixler

**Affiliations:** 1 Radiance Technologies Inc, 310 Bob Heath Dr., Huntsville, AL 35805 USA; 2 Air Force Research Laboratory, 711th Human Performance Wing, Airman Systems Directorate, Bioeffects Division, JBSA Fort Sam Houston, 4141 Petroleum Road, San Antonio, TX 78234 USA

**Keywords:** Computational biophysics, Applied optics

## Abstract

Dynamic, in vivo measurement of the optical properties of biological tissues is still an elusive and critically important problem. Here we develop a technique for inverting a Monte Carlo simulation to extract tissue optical properties from the statistical moments of the spatio-temporal response of the tissue by training a 5-layer fully connected neural network. We demonstrate the accuracy of the method across a very wide parameter space on a single homogeneous layer tissue model and demonstrate that the method is insensitive to parameter selection of the neural network model itself. Finally, we propose an experimental setup capable of measuring the required information in real time in an in vivo environment and demonstrate proof-of-concept level experimental results.

## Introduction

The optical properties of tissue effect a huge variety of biomedical applications, including pulse oximeters, safety standards^1^, deep tissue imaging^2,3^, and photodynamic therapy. Despite this, in vivo measurement of tissue optical properties remains elusive, and published data from ex vivo measurements are varied due primarily to varying tissue preparation and water contents^4^. A primary challenge in any measurement of tissue optical properties is the intrinsically coupled nature of scattering and absorption, and the lack of an analytic solution for light transport for all but the simplest of geometries. Monte Carlo simulations have become the gold standard for light transport in biological media, providing a statistical solution to the forward problem, defined as the simulation of the radiation transport for a given set of optical properties^5–10^. However, due to the relatively long run times and noisy solutions of Monte Carlo simulations, they are problematic for solving the inverse problem and extracting the optical properties for a given measurement of the radiation transport^11^.

Due to a lack of analytical solutions for radiation transport in turbid media, machine learning has been proposed as an approach for inverting Monte Carlo simulations in various frameworks to extract tissue properties^12–14^. Authors have published studies on predicting cancer using least-squares support vector machines^15^, classifying the severity of burns using machine learning and spatial frequency-domain imaging^16^ and a spectroscopic approach to characterizing articular cartilage^17^. The majority of these techniques are applied in reflection using some form of diffuse reflectance^12,16^, but there are a few that are designed in transmission as well^18^; however, none of these approaches lend themselves well to being able to make measurements at high speeds in vivo. The approach presented here differs significantly from other work in that we are focused on extracting optical properties from a single measurement to study dynamic effects with the long term goal of understanding the mechanisms of tissue damage, such as burns. Additionally, we introduce a transformation of the raw simulation data into the statistical moments of the intensity distribution which is a more dense representation of the data that reduces the neural network complexity and increases computational efficiency. This seemingly minor improvement allows our inversion model to span several decades of trade space of optical properties, covering the entire biologically relevant range, while simultaneously allowing index of refraction to be accurately predicted at the same time.

For the purposes of this work, machine learning can be thought of as just the minimization of a cost function over some problem space that is relatively robust to noise due to the large number of random realizations in the training set. The robustness to noisy data makes neural networks very attractive for inverting Monte Carlo simulations. Training can take a long time and require large data sets that require substantial computer resources to generate; however, once the network is trained, execution is fast allowing for real time inversion of dynamic data during experiments. Furthermore, the inputs used are selected to be compatible with standoff in vivo techniques. The simplicity of the network, due to the use of higher order moments as the inputs, reduces the amount of data required for training and increases the speed of execution. These improvements enable our inverse problem to successfully span a much larger parameter space over all reasonable values for biological tissue absorption and scattering while including the index of refraction as an output of our inverse algorithm.

In this paper, we train a deep neural network to invert the Monte Carlo simulation. This is made possible by making use of a GPU accelerated Monte Carlo Multi Layer (MCML) based simulation developed previously^5,19^ to generate the large training sets required for the deep neural network in a relatively short time. The training set used in this work was generated over a weekend on a single desktop computer. We demonstrate that this learning is quite robust to the specific configuration of neural network, indicating that the problem is being successfully generalized as opposed to over fitting of a specific data set, and that it produces very accurate inversion across the range of interest for the values of the optical properties of human skin. Additionally, we propose an experimental setup using streak camera imaging or Compressed Ultrafast Photography (CUP)^20,21^ for making the experimental measurements necessary to extract optical properties in vivo to study dynamic changes in optical properties.

## Methods

**Figure 1:**
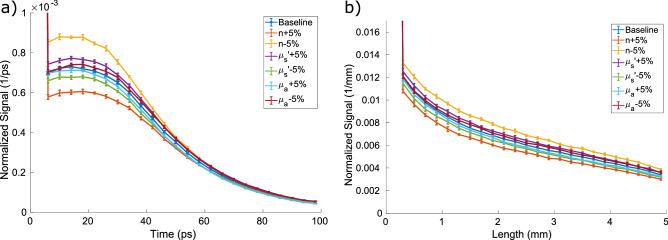
(**a**) Temporal and (**b**) spatial characteristics of a reflected impulse pulse showing the difference for perturbations of the optical parameters. The baseline geometry has $n=1.4$, $g=0.85$, $\mu _{s^{\prime }} = 1\,{\mathrm {mm}}^{-1}$, and $\mu _{a} = 0.1\,{\mathrm {mm}}^{-1}$, which are realistic values for a slab of tissue. The error bars are the standard deviation of 10 independent runs of $10^{6}$ photons each. The large initial point on each plot is large due to Fresnel reflections.

The Monte Carlo model we use is described in full detail in previous work^19^. The core model is essentially a GPU implementation of a traditional MCML model with the scattering probability distribution function (PDF) given by an exponential distribution,

1 $$\begin{aligned} \rho (d) = \mu _{s} e^{-\mu _{s} d} \end{aligned}$$

where $\mu _{s}$ is the scattering coefficient. Anisotropic scattering is handled using the standard Henyey–Greenstein distribution function,

2 $$\begin{aligned} \rho (\cos (\theta )) = \frac{1-g^{2}}{2[1+g^{2}-2g \cos (\theta )]^{3/2}} \end{aligned}$$

where $g= \left\langle \cos (\theta ) \right\rangle$ is the anisotropy coefficient that is typically around 0.9 for tissues. Absorption is treated by attenuating the weight of a photon using a Beer–Lambert equation. Once a photon’s weight is reduced to $10^{-6}$ it undergoes a Russian Roulette process where photons with weights below the threshold have a 1/*n* chance of surviving. To conserve energy, surviving photons are given a new weight of *n* times their old weight and allowed to propagate. Photons are detected when they leave the slab in either the transmission or reflection geometry. The location, direction, weight, and time of arrival of every photon is logged. Representative outputs of this simulation showing the effects of perturbations in the optical properties are shown in Fig. 1.

To generate the training sets, $2.5 \times 10^{5}$ photons are launched at time $t=0$ at $\langle x, y, z \rangle = \langle 0, 0, 0 \rangle$ to simulate the impulse response of the tissue slab. If desired, this impulse response could be convolved with a given detector response and laser pulse to better track with a given experimental setup and detector noise could be added, although these effects are neglected here for simplicity. To confirm the validity of this approach we restrict ourselves to single layer slabs that are nominally 10 mm thick and are effectively infinite size in the transverse plane. We generate optical properties for individual runs of the Monte Carlo simulation by randomly drawing uniform random numbers and restricting them to span the space of values which are most relevant for biological tissues. If $x_{1}$, $x_{2}$, $x_{3}$ and $x_{4}$ are independent uniform random numbers between 0 and 1 then the optical properties for the given run are given by,

3 $$\begin{aligned} n= & {} (n_{{\max }} - n_{{\min }})x_1 + n_{{\min }} \end{aligned}$$

4 $$\begin{aligned} g= & {} (g_{{\max }} - g_{{\min }})x_2 + g_{{\min }} \end{aligned}$$

5 $$\begin{aligned} \log (\mu _{s})= & {} \left[ \log (\mu _{s,{\max }}) - \log (\mu _{s,{\min }}) \right] x_{3} + \log (\mu _{s,{\min }}) \end{aligned}$$

6 $$\begin{aligned} \log (\mu _{a})= & {} \left[ \log (\mu _{a,{\max }}) - \log (\mu _{a,{\min }}) \right] x_{4} + \log (\mu _{a,{\min }}) \end{aligned}$$

where the range of each parameter is given by $1.3 \le n \le 1.6$, $0.5 \le g \le 0.95$, $0.1~{\mathrm {mm}}^{-1} \le \mu _{s} \le 100~{\mathrm {mm}}^{-1}$, and $0.01~{\mathrm {mm}}^{-1} \le \mu _{a} \le 10~{\mathrm {mm}}^{-1}$. These ranges were chosen to cover the span of values measured for various biological tissues. A log scale spacing was chosen for the scattering and absorption coefficients to better sample the full space of values since they span several decades of values. For both training and testing, the anisotropy and scattering coefficient are combined into the reduced scattering coefficient,

7 $$\begin{aligned} \mu _{s}{^{\prime }} = (1-g) \mu _{s} \end{aligned}$$

Light transport in tissue is dominated by diffusion effects, thus the solutions tend to be largely dependent on $\mu _{s}{^{\prime }}$ instead of *g* and $\mu _{s}$ directly. Therefore, developing a network that is sensitive to only the reduced scattering coefficient tends to converge towards high accuracy more quickly, so we opted for this approach.

**Figure 2:**
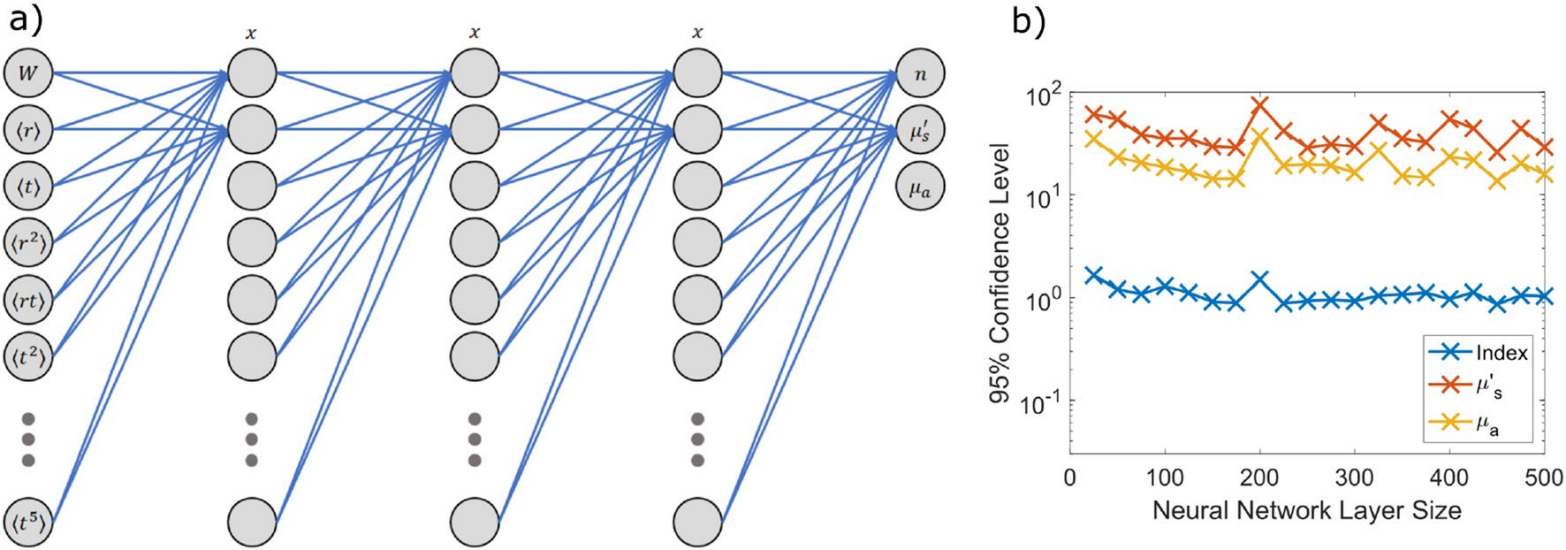
(**a**) Schematic of the fully connected neural network used to solve the inverse problem where *x* is varied to study the impact of network size on accuracy (**b**) The dependence of prediction accuracy on network layer size demonstrating that the prediction accuracy of the approach is not terribly sensitive to the size of the network used. This illustrates that this approach is a general one that is robust to small changes in network parameters. *n* is the index of refraction, $\mu _{s}{^{\prime }}$ is the reduced scattering coefficient defined in Eq. (7), and $\mu _{a}$ is the absorption coefficient. The statistical moments used for the inputs to the model are defined by Eq. (8).

## Results and discussion

One of the challenges of fully connected neural networks like the one used here is that the number of weights grows dramatically with increased layer size. As a result, it is highly desirable to have the input vector into the network be a dense representation of the problem. In principle, the raw spatial-temporal profiles from the Monte Carlo simulation could be input into the neural network and allow the training to determine what is important; however, this would require a much larger network and training set. Instead of using binned data similar to that shown in Fig. 1, we use the spatial-temporal moments of the irradiance distribution as the inputs. There is nothing to break rotational symmetry in the problem so we can reduce the dimensionality of our results from *I*(*x*, *y*, *t*) to *I*(*r*, *t*). These moments can be computed directly from the raw output of the Monte Carlo simulations by

8 $$\begin{aligned} \langle r^{\alpha } t^{\beta } \rangle = \frac{\sum _{i} w_{i} (r_{i}-\langle r \rangle )^\alpha (t_{i} - \langle t \rangle )^\beta }{W} \end{aligned}$$

Here, $W = \sum _{i} w_{i}$, $\langle r \rangle = (\sum _{i} w_{i} r_{i})/W$, and $\langle t \rangle = (\sum _{i} w_{i} t_{i})/W$, $r_{i}$ is the radial distance that the *i*th photon exited the medium, and $t_{i}$ is the time that the *i*th photon exited the medium. Eq. (8) is only valid for $\alpha > 1$ and $\beta > 1$ with the lower order moments defined by *W*, $\langle r \rangle$, and $\langle t \rangle$. For this work here, we use the first 5 orders of moments, consisting of 21 parameters, as inputs into our Monte Carlo simulation.

**Figure 3:**
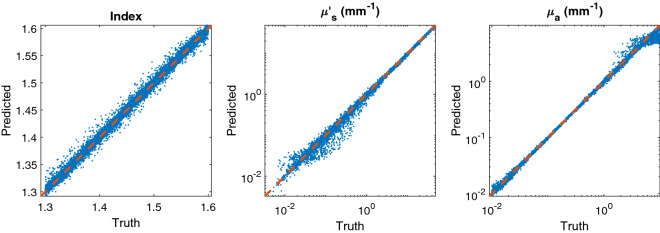
Prediction versus truth for a [21, 150, 150, 150, 3] fully connected neural network illustrating the robustness of this approach over the majority of the problem space. The dashed red line is the line $y=x$ which would correspond to a perfect prediction.

Due to the dense representation of the problem offered by the statistical moments, we have decided to utilize a very simple fully connected neural network. We will demonstrate that even this simple neural network will provide us sufficient accuracy to continue with it moving forward. To determine viability of the approach we tested 5 layer fully connected networks with variable size hidden layers but with 21 input moments, $\alpha + \beta \le 5$ in Eq. (8), and the 3 outputs, *n*, $\mu _{s}{^{\prime }}$, and $\mu _{a}$, so that the overall size of the network was [21, *x*, *x*, *x*, 3] as shown schematically in Fig. 2a. *x* was varied between 25 and 500 and the networks were all trained on the same training set and then the prediction errors were determined by comparing the known values, truth, to those predicted by the neural network. The inputs are all normalized to values between 0 and 1 by using

9 $$\begin{aligned} {\tilde{x}} = \frac{X-{\min }(X)}{{\max }(X)-{\min }(X)} \end{aligned}$$

where *X* can represent *n*, ${\log }_{10}(\mu _{s}{^{\prime }})$, and ${\log }_{10}(\mu _{a})$ for input into the model for numerical stability and the inverse of this process is applied on the output. For a measure of error we use the standard error

10 $$\begin{aligned} E = 100\% \times \frac{ \left| X_{\mathrm {truth}} - X_{\mathrm {predicted}} \right| }{ X_{\mathrm {truth}} } \end{aligned}$$

where *X* can represent *n*, $\mu _{s}{^{\prime }}$, or $\mu _{a}$ respectively. We define the $95\%$ confidence level as the value of the error where $95\%$ of the data points have less error.

For training and testing the neural networks, a total set of 305,598 independent Monte Carlo runs were generated. 10,000 randomly selected points were set aside as a testing set and the rest of the set was used for training. The networks were never exposed to the testing set data points until training was complete and were used exclusively for measuring the accuracy of the model. The training was allowed to run for 5000 epochs in each case to ensure convergence. Additionally, we define the $95\%$ confidence level as the value of the error where $95\%$ of the test data points have less error, and plot these results as the red line in Fig. 2b. These results demonstrate that we can expect a prediction accuracy of about $1\%$ in *n*, $30\%$ in $\mu _{s}{^{\prime }}$, and $15\%$ in $\mu _{a}$ across the vast majority of the parameter space. These are more than sufficient for our purpose considering these parameters vary by approximately a factor of 5 in the literature, typically attributed to variations in tissue and tissue preparation^4^. Moving forward we will use a network of size [21, 150, 150, 150, 3] because this gives good accuracy and is small enough that over fitting is not a concern. As shown by Fig. 2b this is more of an arbitrary choice than a necessary one as the results are similar over a large range of network sizes.

The computational requirements of this approach are overall very modest and dominated by the generation of the training set. This required a desktop computer utilizing an NVIDIA RTX2080 Ti GPU and an Intel i7-7820X CPU running Monte Carlo simulations over a weekend. The training of the neural network, due to its simplicity, only took around 15 min on the same computer. Execution of a single optical properties prediction from the neural network is very quick, requiring 53 ms to compute a batch size of 10,000 for an average execution time of 5.3 $\upmu$s for a single point. This statement should have the caveat that unless a real time operating system is used, these single point times wouldn’t likely be achievable in practice due to uncertainty in the code execution on such timescales.

**Figure 4:**
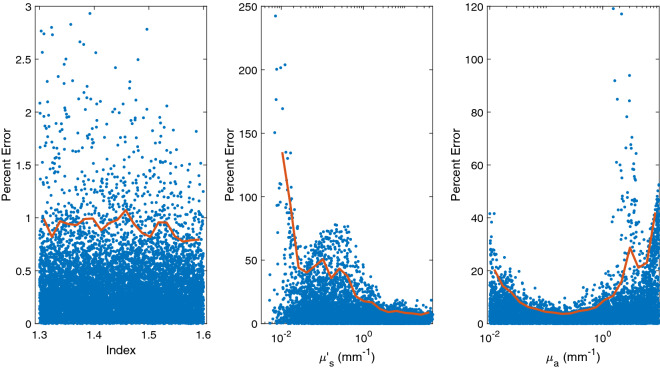
Prediction error for a [21, 150, 150, 150, 3] fully connected neural network illustrating the accuracy of the method across the problem space. The red line is the $95\%$ error level. The errors are large when scattering and absorption are small because the light transport does not depend strongly on these variables in those cases and when the absorption is large because a lot of the light is absorbed in the medium and never reaches a detector. In these three situations a large error is expected and represents a limitation of the physics of the problem more so than a limitation of this technique.

The accuracy of the neural network prediction is shown for each parameter in Fig. 3, where the neural network prediction is plotted against the true value for that point. The individual blue dots are individual points from the testing set and the dashed line $y=x$ would signify a perfect prediction. For reference, typical measured skin tissue in the visible has a $\mu _{s}{^{\prime }}$ from 1 to $10~{\mathrm {mm}}^{-1}$ and $\mu _{a}$ from 0.1 to $10~{\mathrm {mm}}^{-1}$^4^. In every case you can see that the prediction is highly correlated but the network performs better in some regions than others. This illustrates the complexity of solving the inverse problem for light transport in tissue. For low scattering, the irradiance in reflection is not highly dependent on the value of the scattering coefficient, so the accuracy of the inverse prediction is lower. Absorption is even more complicated because low values of absorption are similarly hard to predict as low values of scattering, but high values are also difficult because so much of the light is attenuated in the tissue and never makes it to a detector. As a result, this degradation in accuracy is more of a limitation on the physics of the problem than it is a limitation on any one way of solving the inverse problem as all methods would be expected to suffer from lower accuracy in these regimes. These arguments compound further when discussing multi-layer or heterogeneous samples, elucidating the fundamental difficulty of the problem. Conversely, the accuracy is more or less consistent across the full space for the index of refraction. This is because the light transport solution depends substantially on the index of refraction due to both initial and internal Fresnel reflections, which can be seen in the large value of the first data point (not shown on scale) in Fig. 1. The variability in the prediction accuracy over the problem space is quantified further in Fig. 4 which shows the standard error for each data point as well as the line of $95\%$ certainty.

**Figure 5:**
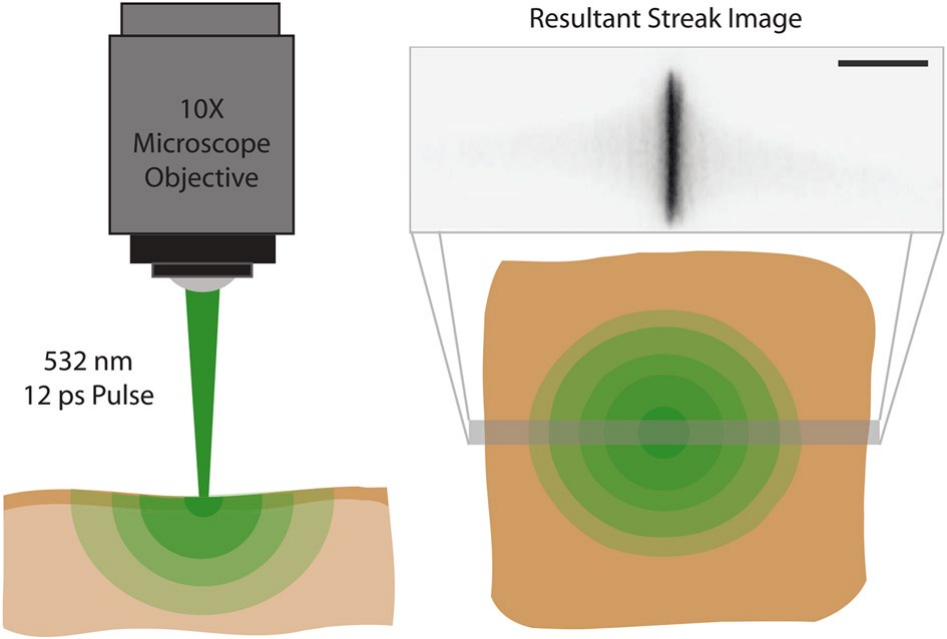
Schematic of the proposed experimental realization for the machine learning estimation of tissue optical properties. The gray line on the right corresponds to the section of tissue that would be imaged to the entrance slit of the streak camera. Scale bar is $150~{\upmu }$m.

Experimental realization of the theory presented in this paper can be achieved through the use of a high dynamic range streak camera. For proof-of-concept experiments, we employ a Hamamatsu C7700 streak camera (Hamamatsu, Japan) to acquire back scattered photon distributions with picosecond time resolution. Tissue samples were placed on the sample stage of an Olympus iX-73 microscope and imaged with a 10$\times$ objective (Olympus, USA). A 6-ps, 532 nm laser pulse (Attodyne ALP-10) was coupled into the rear port of the microscope, directed to the sample stage via a 50/50 beamsplitter (Thorlabs, USA) and the back scatter was relayed to the streak camera. The streak camera has a temporal resolution limit of 2 ps. The use of a commercial microscope is not critical for this concept, and numerous other collection geometries could be employed to control field of view and spatial resolution. For homogeneous slabs such as the synthetic tissue phantoms used here, the rotational symmetry assumed for our Monte Carlo simulations is analogous to the single spatial dimension provided by the streak camera. In this case, we capture a full line of signal, centered on the laser spots location on the tissue. Fig. 5 provides a simple cartoon illustrating our collection geometry, along with a representative streak.

Initial experiments were conducted using synthetic tissue samples with known optical properties. Syndaver tissue coupons (Syndaver, USA) with Asian pigment levels were imaged with the streak camera microscope described above, and the optical properties of these samples were previously validated by NIST using a dual integrating sphere system^22,23^. As each row of the kymograph from a streak camera provides a photon distribution for a point in time, where temporal resolution is controlled by the voltage ramp applied to the streak tube, we can resolve the diffusion and attenuation of photons interacting with a tissue sample by evaluating each row of pixels in the image. A video of the event can be created from a streak image by 180$^\circ$ rotation of each row of pixels around the center point, where each successive row of pixels would become the next frame in the video. Initial analysis of the streak data shows an interaction time of $63.5 \pm 4.93$ ps, which matches the simulation data well considering the noise floor of the streak camera. Current efforts are focused on the pre-processing required for this experimental data to be evaluated by the neural network, with particular focus on addressing difficulties in identifying time zero in the streak. Future studies will be preformed on synthetic tissue phantoms with controlled optical properties ($\mu _{a}$ and $\mu _{s}{^{\prime }}$) to validate the sensitivity of the proposed methods to a biologically relevant range of optical properties. Beyond this, Compressed Ultrafast Photography (CUP) is a logical extension of ultrafast imaging, providing a means to acquire the full spatial distribution of photons over time, and could be further explored^20,21,24^.

## Conclusion

To conclude, we have demonstrated that it is possible to train a neural network to solve the inverse of a Monte Carlo simulation for extraction of optical properties for a single homogeneous layer using the spatio-temporal response of the tissue in the reflection geometry. We have quantified the errors associated with this inversion and have demonstrated that they are small enough to produce useful results. Additionally, we have proposed an experimental measurement of the exact spatio-temporal response needed for this that is capable of acquiring this information in a single optical pulse operating at 100 Hz, opening the door to studying dynamic tissue optical properties in an in vivo setting. Overall, a combination of inverting Monte Carlo simulations and the experiment proposed will enable more accurate measurements of tissue optical properties that can be performed non-invasively in real time.

## Data Availability

The datasets generated during and/or analysed during the current study are available from the corresponding author on reasonable request.
